# CircRNP complexes: from nature to design

**DOI:** 10.1093/jmcb/mjad006

**Published:** 2023-01-31

**Authors:** Stephen Sukumar Nuthalapati, Corinna Jessica Ulshöfer, Albrecht Bindereif

**Affiliations:** Institute of Biochemistry, Justus Liebig University of Giessen, 35392 Giessen, Germany; Institute of Biochemistry, Justus Liebig University of Giessen, 35392 Giessen, Germany; Institute of Biochemistry, Justus Liebig University of Giessen, 35392 Giessen, Germany

Circular RNAs (circRNAs) have recently been established as a new and large class of noncoding RNAs, conserved in all eukaryotes investigated. However, functional and mechanistic investi-gations lag behind the rapid progress in circRNA expression studies, and no uniform function has emerged so far. Here, we focus on circRNA–protein (circRNP) complexes (circRNPs), since proteins associated with natural circRNAs likely guide us in the search for physiological functions of individual circRNAs. First, approaches for how to systematically search for circRNA-interacting proteins in natural circRNPs will be summarized, including some experimental considerations important for good scientific practice. Second, building on natural protein-sponging circRNAs, we introduce the concept of designer circRNAs functioning as specific protein sponges, using the heterogeneous nuclear ribonucleoprotein L (hnRNP L) and IMP3 proteins as two examples of classical RNA-binding proteins. Third, we discuss the general implications of this concept, in particular how it can be applied as a new way to intervene with RNA-based gene-regulatory networks. In sum, designer circRNAs sponging specific proteins open up exciting new perspectives in molecular medicine and on therapeutic options to treat human disease.

## CircRNAs: biogenesis, abundance, functional question

CircRNAs were first discovered in 1976 as important plant–pathogenic viroid RNAs, revealed in their circular configuration by electron microscopy (EM; Sänger et al., 1976). Similarly, circRNA species were EM-visualized in cytoplasmic RNA from mammalian cells (Hsu and Coca-Prados, 1979), and the genome of the hepatitis delta virus was identified as a circRNA (Kos et al., 1986). Some more, sporadic examples of circRNAs from various organisms and RNA classes appeared over the years (reviewed in Wilusz, 2018; Kristensen et al., 2019; Chen, 2020; Yang et al., 2022); until ∼10 years ago, thanks to systematic sequencing-based searches for noncoding RNA, circRNAs were rediscovered as a large class of noncoding RNAs in all higher eukaryotic species analyzed (Salzman et al., 2012; Jeck et al., 2013; Memczak et al., 2013). This abundant and widespread class of circRNAs is generated from nuclear pre-messenger RNAs (pre-mRNAs) by a special type of alternative splicing (Starke et al., 2015). Thereby, instead of splicing to the normal linear mRNA, single exons or several adjacent and spliced exons are excised in a circular configuration. Like conventional mRNA, such exonic circRNAs are exported to the cytoplasm, where they accumulate, due to their unusual stability.

Functionally, however, circRNAs have remained largely enigmatic until today (Hentze and Preiss, 2013), with few exceptions.

First, one of the best-characterized circRNAs, ciRS-7 (or cdr1as) is abundantly expressed in the brain, conserved, and carries > 70 miR-7-binding sites per circRNA in humans, clearly functioning in miRNA-specific sponging (Hansen et al., 2013; Memczak et al., 2013). Similarly, the testis-specifically expressed SRY circ-RNA in mouse, with 38 binding sites for miR-138, appears to act as a miRNA sponge (Hansen et al., 2013). However, no other circRNA with such large numbers of unique miRNA sites is known. Therefore, considering the stoichiometric ratio of circRNA to miRNA, it is unlikely that miRNA sponging represents a common or widespread circRNA function.

Second, sponging of RNA-binding proteins (RBPs) by circRNAs has been documented in a few cases, first for circMBL, a circRNA in *Drosophila* derived from the second exon of the muscleblind (*MBL*) gene expressing the MBL splicing factor (Ashwal-Fluss et al., 2014). Linear-versus-circular RNA processing of the *MBL* gene in fly appears to be autoregulated in a feedback loop, based on binding of the MBL protein product to flanking intron regions around exon 2, thereby shifting *MBL* pre-mRNA processing from linear mRNA to the circRNA product, which in turn binds to MBL protein (for other reported circRNA-mediated protein sponges, see Liu et al., 2019; Tsitsipatis et al., 2021).

It remains a major question in the circRNA field, whether there exists a common unifying function or mechanistic principle for circRNAs. Based on our current knowledge, this appears not to be the case, and more likely, natural circRNAs cover a diverse functional spectrum. In fact, a fraction of circRNAs may also represent background noise of gene expression, considering the very low expression levels of many circRNAs over the predominant linear mRNA isoforms. Because of the low abundance of many circRNAs, particular care has to be taken to follow stringent guidelines and good scientific practice in circRNA-focused experimental work (Nielsen et al., 2022). Nevertheless, other, mostly hypothetical functions should be considered for natural circRNAs (Hentze and Preiss, 2013), for example, antisense activities, scaffolding, structural, or allosteric regulator roles, or functions in peptide and protein translation (Yang et al., 2022). In addition to systematic searches and screening for new functions of natural circRNAs, an alternative approach can be chosen: designing or *in-vitro*-evolving circRNAs with predicted and optimized functions to be used for modulation of gene expression (for examples, see Jost et al., 2018; Schreiner et al., 2020; Pfafenrot et al., 2021). Applications of synthetic designer circRNAs can range from basic biology to therapeutic medicine, and in turn, will stimulate investigations into their natural existence.

## CircRNA–protein interactions: from natural circRNPs to artificial circRNA sponges, design and experimental principles

How to search for and investigate natural circRNPs? Numerous cases have been reported in the last few years (summarized by Zhou et al., 2020). For early examples of specific circRNA–protein interactions, see Ashwal-Fluss et al. (2014), Conn et al. (2015), or Schneider et al. (2016). In principle, there are three general approaches that may be used, separately or in combination. Since a certain circRNA contains the same RNA sequence and protein-binding sites as the corresponding linear mRNA, one has to consider in each case how to distinguish between these two RNA configurations. For example, circRNA-specific primer pairs in reverse transcription–polymerase chain reaction (RT–PCR), or circ-junction spanning sequence reads in crosslinking–immunoprecipitation (CLIP) assays, may be used.

First, gradient sedimentation (glycerol- or sucrose-based) separates macromolecular complexes by size and therefore allows the direct comparison of free and protein-bound RNAs. We have used this approach for identifying circRNPs present in cellular extracts and evaluating the shift in gradient mobility of specific circRNAs and their circRNPs. The extent of the shift yields initial information on the size of the circRNPs and the total molecular mass of the associated proteins (Schneider et al., 2016).

Second, as a protein-based approach, circRNPs can be selected by co-immunoprecipitation, which in combination with RNA-sequencing (RNA-seq) identifies subgroups of circRNPs associated with a protein of special interest. For example, we have revealed a group of circRNAs with the IMP3 protein bound as a stable component (Schneider et al., 2016). In addition, information derived from CLIP assays available for many RBPs may be integrated in such analyses.

Third, a circRNA-based approach has been applied in a few instances, using affinity selection of circRNPs, using biotin-labelled circRNAs and streptavidin agarose, or biotin selection with antisense oligonucleotides (Pandey et al., 2020; Tsitsipatis et al., 2021). In these cases, protein components can be identified by subsequent mass spectrometry and should be validated by immunoprecipitation and circRNA-specific RT–PCR.

When designing new circRNAs for protein sponging, we may be guided by natural circRNAs, in particular the *bona fide* miRNA sponges, each of which contains oligomeric binding sites for a single specific miRNA (see above). This principle can be directly transferred to protein-sponging circRNAs: stoichiometry of circRNA-binding sites on the circRNA and cellular concentrations and localization of the target protein, as well as the affinity of circRNA–protein interaction, are most important parameters for an efficient circRNA-based protein sponge (for an example of quantitations of overexpressed circRNA sponges and the endogenous hnRNP L target protein, see Schreiner et al., 2020).

Three additional aspects should be taken into consideration in the design of protein-sponging circRNAs.

First, for selecting an optimal binding site for an RBP, we should take into account the molecular structure of the specific RNA–protein interaction: does the RBP operate through only a single RNA-binding domain, or are multiple domains involved in RNA recognition? The latter case of multidomain RBPs is quite common. For example, many hnRNP proteins carry several RRM domains (such as the four RRMs in hnRNP L), or another classical RBP, IMP3, recognizes RNA through two RRMs and four KH domains (Korn et al., 2021). Therefore, for multidomain RBPs, optimal, high-affinity binding sites can be composed of an array of several short binding modules with appropriate, optimal spacing in between. To design perfect binding sites requires knowledge of the short RNA-binding elements, often 4–6 nucleotide sequences derived from CLIP or other global studies (for examples, see Hafner et al., 2010; Ray et al., 2013; Dominguez et al., 2018). Alternatively, initial SELEX-based strategies may generate such high-affinity binding sequences (for an example, see Schneider et al., 2019).

Second, the optimal binding site, as a single copy or as an oligomerized array, has to be integrated into a circRNA backbone. The design and resulting circRNA size determines how it can be generated, whether by T7-transcription and enzymatic ligation by T4 RNA ligase (mostly for short circRNAs of less than ∼150 nucleotides; see Breuer and Rossbach, 2020), or by ribozyme-mediated circRNA expression (short or long circRNAs; for the so-called PIE and Tornado systems, see Wesselhoeft et al., 2018; Litke and Jaffrey, 2019). Both kinds of *in-vitro*-generated circRNAs are usually purified and can be functionally studied initially by *in vitro* systems in extracts, or following transfection in cell culture, and ultimately in animal models. An obvious advantage thereby is that circRNA amounts and concentrations can be titrated, and dosis-dependent effects help in the functional analysis of gene expression. On the other hand, the circ-RNA expression by the Tornado system in cell culture, which involves a ribozymatic excision and an RtcB tRNA-ligase step, allows dramatic overexpression of both small and large circRNAs, up to millimolar levels in the cell, corresponding to >10^6^ copies per cell (Litke and Jaffrey, 2019; Schreiner et al., 2020). Finally, functional studies have to consider that transfected circRNAs distribute between cytoplasmic and nuclear space, whereas Tornado-expressed circRNA, which follow the natural biogenesis pathway, localize mostly to the cytoplasm.

Third, the design of artificial circRNAs should also take into consideration their RNA structure and topology. In particular for smaller circRNAs, RNA-structure prediction programs can be applied. Experimentally, to assess the efficiencies of protein sponging, it helps to directly compare the circRNA with its linear counterpart of identical sequence. Such quantitative circRNA–protein binding assays would reveal the importance of the sequence length of sponges, the relevance of oligomerized motifs, and the potential effects of circRNA topology. Finally, when quantitating circRNA–protein interaction in cells, it is advisable and much more informative to express binding efficiencies relative to the input (as a percentage of bound circRNA), instead of comparing enrichment factors (fold-changes) relative to an unspecific control (for IMP3, see Schneider et al., 2016).

## Designer circRNA-based protein sponges: two examples

These principles are illustrated in the following with experimental data from two exemplary cases of multidomain RBPs, IMP3 and hnRNP L.

**Figure 1:**
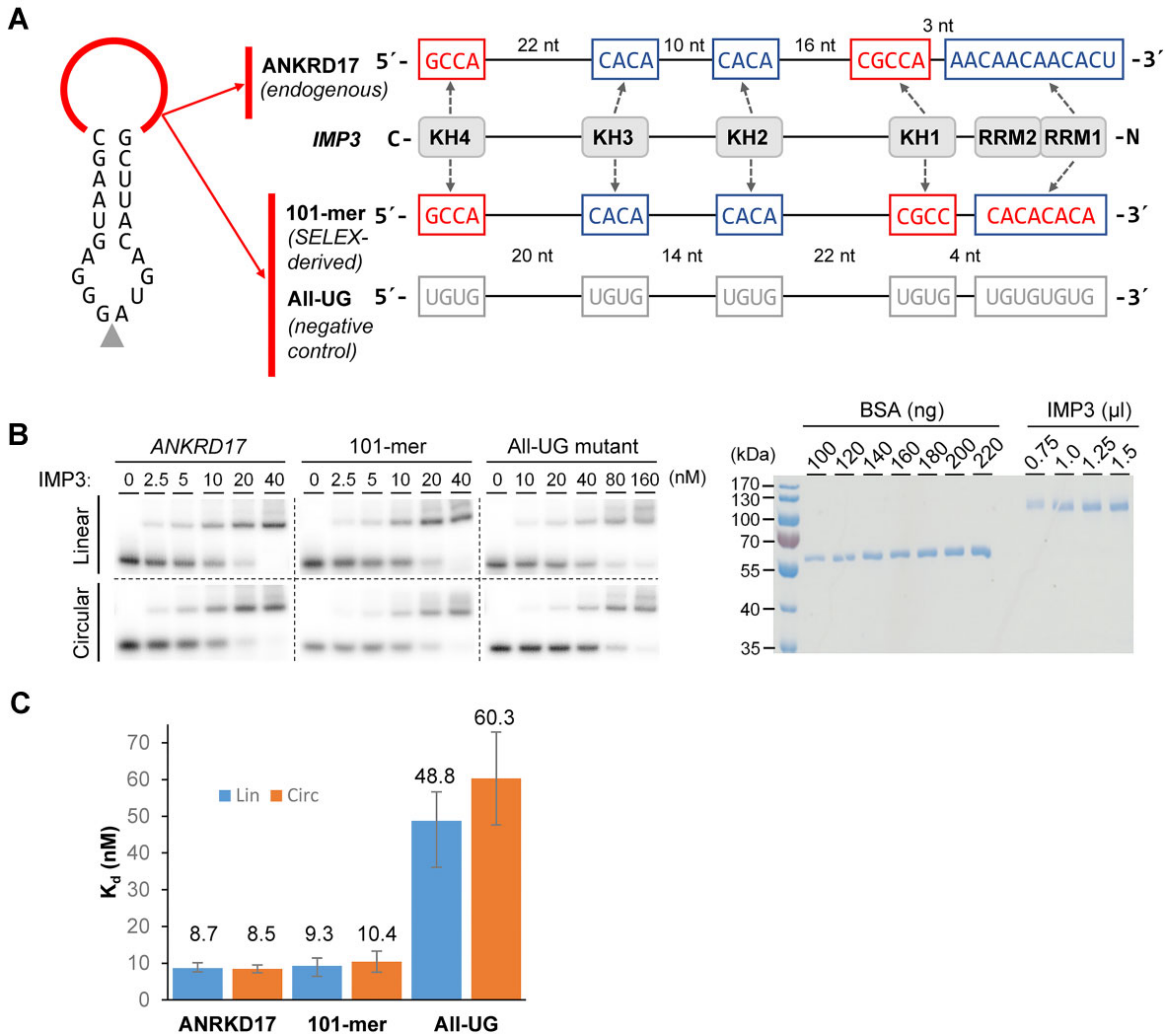
IMP3 protein sponging *in vitro*: comparing circular and linear RNA binding. (**A**) Design of small circRNAs (ANKRD17 and 101-mer, with all-UG as the negative control) for sponging of IMP3 protein *in vitro*. Each of these circRNAs is based on a 20-nucleotide stem-loop, with the following sequences inserted (in red; *in vitro* circularization site indicated by arrow). ANKRD17 circRNA contains the high-affinity IMP3 binding array of 121 nucleotides, which was found in the natural circRNA that is processed from exon 29 of human *ANKRD17* gene (Schneider et al., 2016). This general RNA-recognition code of IMP3 protein has been derived by SELEX-seq and iCLIP data integration (Schneider et al., 2019). The 101-mer circRNA carries a SELEX-derived, optimized, and validated IMP3 binding array of 101 nucleotides (Schneider et al., 2019), and the all-UG circRNA serving as a negative control, where the binding modules were each mutated to UGUG (sequences of the binding elements and of the IMP3 RNA-binding domains on the right). (**B**) Binding of recombinant IMP3 protein to circRNA. Recombinant GST-/His-tagged IMP3 protein was purified from *Escherichia coli* cells by Ni-NTA agarose chromatography, followed by sodium dodecyl sulfate–polyacrylamide gel electrophoresis and Coomassie staining (to the right). Samples were analyzed after dialysis of the combined eluates (0.75–1.5 μl) and quantitated by comparing with known amounts of BSA (100–220 ng). Linear and *in vitro*-ligated ^32^P-labelled RNAs (ANKRD17, 101-mer, all-UG) were gel-purified and incubated with recombinant IMP3 protein (0–40 or 0–160 nM range, as indicated; 5 nM RNA), and binding was analyzed by EMSA (to the left). (**C**) Determination of dissociation constants for IMP3 protein interactions with circular or linear RNAs (ANKRD17, 101-mer, all-UG), based on the data from the EMSA assays in **B**. *K*_d_ values are depicted with standard deviations from triplicate experiments.

First, the domain structure of the multifunctional IMP3 protein consists of two N-terminal RRM domains, followed by four KH domains; how these multiple domains collaborate in RNA recognition has recently been studied by SELEX and structural approaches, revealing a cluster of five distinct RNA elements spread over ∼100 nucleotides (Schneider et al., 2019). This validated high-affinity RNA-binding array of IMP3 had been identified in a subgroup of natural circRNAs that are all IMP3-associated (Schneider et al., 2016). We used this array as a synthetic and optimal consensus sequence of 101 nucleotides, inserted in a 20-nucleotide stem-loop, to biochemically evaluate its IMP3 interaction in a minimal circRNA context (Figure 1A). By *in vitro* RNA–protein interaction assays, we compared directly circular and linear configurations of this sequence array, using band shift assays with recombinant IMP3 protein and *K*_d_ measurements (Figure 1B and C). We found that both forms bound to IMP3 protein with similar affinities of ∼9.3 and 10.4 nM (linear vs. circular SELEX-derived 101-mer), comparable to a natural, *ANKRD17*-derived sequence (8.7 and 8.5 nM); in contrast, in the negative control sequence with mutated elements, IMP3-binding affinities were strongly reduced (all-UG; 48.8 and 60.3 nM). Although the binding affinities of this particular sponge for IMP3 protein are very similar for circRNA vs. corresponding linear isoform, this may differ in either direction for other examples. Note that the effectiveness of sponge circRNAs for *in vivo* applications is determined not only by protein-binding affinity but also by several other factors, such as stability, cellular localization, and protein-binding specificity.

**Figure 2:**
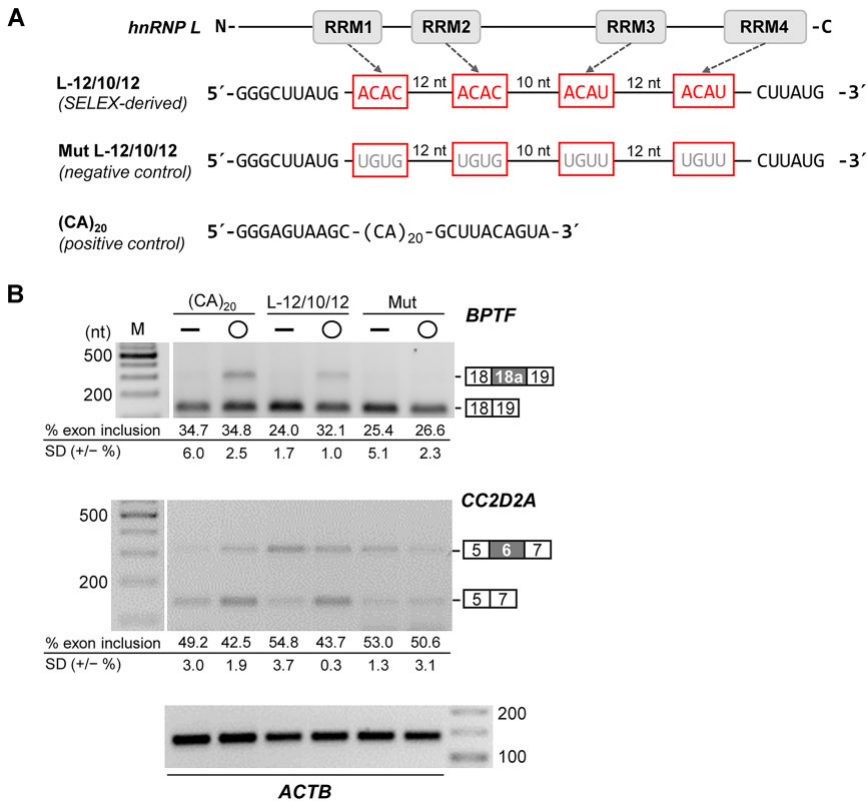
Designer circRNA-based hnRNP L sponges modulate alternative splicing in HeLa cells. (**A**) Design and sequences of small circRNAs for hnRNP L protein sponging *in vivo*: L-12/10/12, mut L-12/10/12 as a negative control, and (CA)_20_ as a positive control. L-12/10/12 is a 65-nucleotide hnRNP L-binding array of four CA-rich elements with optimized spacing, derived from a SELEX study (unpublished data); in the negative control, the elements are mutated to UGUG/UGUU; the (CA)_20_ sequence is inserted into the 20-nucleotide stem-loop (as shown in Figure 1A) and has previously been validated to bind to hnRNP L with high affinity (Schreiner et al., 2020). The specific arrangement of multidomain recognition of individual CA-elements by the four RRM domains of hnRNP L is hypothetical. (**B**) Modulation of alternative splicing of two known L target genes, where hnRNP L acts as a repressor (*BPTF*) or activator (*CC2D2A*). L-12/10/12 RNA, or as controls the mutant version (Mut) or (CA)_20_, either in linear or circular configuration (— or ◯), were transfected in HeLa cells (1 μg per transfection and 10^6^ cells). After 2 days, alternative splicing of *BPTF* and *CC2D2A* was assayed by RT–PCR, including quantitation of exon inclusion levels (three biological replicates, including standard deviations, in %), with β-actin serving as a loading control. M, marker (in nt).

Second, hnRNP L, another multidomain RBP of the hnRNP family, with four RRM domains, functions as a global splicing regulator, activating or repressing specific alternative splicing targets (Hung et al., 2008). We had initially identified the RNA-binding specificity of hnRNP L for certain CA-rich sequence elements, based on a SELEX approach (Hui et al., 2005). Using iCLIP and RNA sequencing, we also addressed the distinction between splicing repressing and activating activities of hnRNP L (Rossbach et al., 2014; for a comprehensive list of repressor and activator targets, see Supplementary Table S1 in Schreiner et al., 2020). Recently, we refined our SELEX analysis, using a longer, *N*_40_-degenerate sequence, which revealed distinct spacing preferences between individual short CA-rich elements (unpublished data; summarized in Figure 2A as a hypothetical model of the interaction of four CA-elements with the four RRMs of hnRNP L). On that basis, a short circRNA, L-12/10/12, was designed and synthesized; the four CA-elements were point-mutated to UG-elements for the specificity control, mut L-12/10/12 (Figure 2A). Specific hnRNP L binding was confirmed (data not shown), as recently described for other short hnRNP L circRNA sponges (Schreiner et al., 2020).

To assess the functionality of the new, optimized L-12/10/12 circRNA sponge in splicing modulation, the circRNA (or the linear version, as a control and for comparison) was transfected in HeLa cells, followed by RT–PCR assays of alternative splicing of two known hnRNP L target genes, *BPTF* and *CC2D2A*, where hnRNP L acts as a repressor or activator, respectively, of a specific alternative exon (Figure 2B): *BPTF* exon 18a (NM_004459) inclusion increased after expression of the L-12/10/12 sponge, whereas *CC2D2A* exon 6 (NM_001378617) inclusion decreased (compare L-12/10/12 ◯ and mut ◯). Comparable effects were observed for another short circRNA sponge (CA)_20_ (as a positive control), as previously described by Schreiner et al. (2020), but not for the negative control with the mutated hnRNP L binding sites. Note that the linear version of these sponges was consistently less active in splicing modulation, likely due to decreased stability in cells, or differences in hnRNP L binding by circular and linear RNAs. We conclude that L-12/10/12 circRNA functions as a minimal hnRNP L sponge, comparable to (CA)_20_ circRNA.

## CircRNAs designed as protein sponges: a new component in RNA-regulatory networks

**Figure 3:**
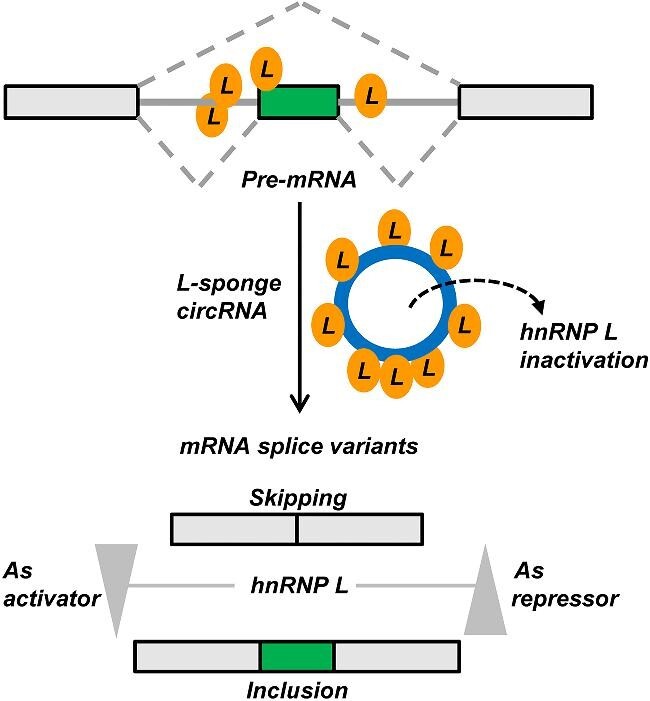
General concept of alternative splicing modulation by circRNA-based sponges: hnRNP L as an example. hnRNP L regulates exon skipping and inclusion, acting as either a splicing activator or a repressor. These splicing decisions can be modulated by sponge circRNAs carrying multiple CA-repeat or certain CA-rich sequences, which bind to multiple hnRNP L molecules with high affinity, thereby functionally inactivating them. This results in a shift in the ratio of splice isoforms (skipping/inclusion), depending on whether hnRNP L acts as a repressor or an activator. This concept can in principle be applied to any splicing-regulatory RBP, for which high-affinity binding motifs are known.

The general principle of designer circ-RNAs acting as protein sponges (Figure 3) has been established for two RBPs, hnRNP L and IMP3 (Schreiner et al., 2020; data shown above in Figures 1 and 2). A circRNA specific for a certain RBP is designed, carrying single or multiple high-affinity binding sites for the RBP; introducing this circRNA into cells (or suitable extract systems) in stoichiometrically sufficient quantities results in functional depletion of the RBP and corresponding defects. For example, for alternative splicing factors, the splicing patterns of the target pre-mRNAs will change, similar to standard RNAi-induced knockdown of the RBP (for a detailed, RNA-seq-based comparison of standard RNAi knockdown and circRNA-mediated sponging of hnRNP L, see Schreiner et al., 2020). Sponging interferes directly at the protein level and relies on the high-affinity binding of an RBP to a circRNA, whereas RNAi acts at the mRNA level and critically depends on the sequence specificity of the siRNA for a certain mRNA. In parallel, it should also be possible to alter other RBP functions by circRNA sponging, such as other RNA-processing activities, RNA stability, cellular localization, or translation. In sum, circRNA-based protein sponging represents a new principle to modulate gene-regulatory networks by a designer noncoding RNA.

How common this principle of circRNA-based protein sponging exists in nature remains still unknown. The autoregulation of MBL expression provides an example (see above), where alternative splicing generates linear and circular isoforms, although the exact circRNA–protein stoichiometry had not been investigated in that case. The classical multidomain-RBP IMP3 may be similarly integrated in such circRNA networks, since a subclass of natural circ-RNAs has been identified that specifically binds to IMP3 (Schneider et al., 2016), and at the same time, IMP3 is very often found associated with 3′-UTR regions, based on iCLIP analysis (Schneider et al., 2016, 2019). Crosstalk between 3′-UTR and circRNAs therefore appears as a plausible hypothesis, for example in the generation of alternative 3′-UTRs.

In conclusion, artificial designer circ-RNAs (here with protein-sponge functions) have been developed as a new principle of interfering agents. Further investigations on the development and use of circRNAs for disease therapies have to consider the potential immunogenicity of newly added RNA agents *in vivo*, the cell-type specificity of such effects, and the impact of RNA modifications (see Liu et al., 2022, and references therein). This will likely result in new applications in molecular medicine, such as new RNA drugs and therapeutic options to treat human disease, as already suggested by Bohjanen et al. (1996). At the same time, such studies should also catalyze searches for physiological functions of natural circRNAs.

*[Supplementary material is available at Journal of Molecular Cell Biology online. We thank Tim Schneider and all other lab members for helpful discussions. Funding is acknowledged from the Deutsche Forschungsgemeinschaft (RTG 2355; project Bi 316/18-1 and 18-2 within SPP 1935 to A.B.) and from the European Union’s Horizon 2020 research and innovation programme under the Marie Sklodowska–Curie grant agreement No 721890 (CircRTrain; to A.B.). A.B. filed a patent application on the use of designer circRNAs as protein sponges (EP 19208168.5, `Circular RNAs and uses thereof for inhibiting RNA-binding proteins'; patent pending). S.S.N. and C.J.U. designed and carried out experiments; A.B., S.S.N., and C.J.U. wrote the manuscript.]*

## Supplementary Material

mjad006_Supplemental_File
