# Supplementary material for: CircRNP complexes: from nature to design
Source: J Mol Cell Biol. 2023 Jan 31;15(1):mjad006. doi: 10.1093/jmcb/mjad006 (PMC10234438; doi:10.1093/jmcb/mjad006)
Supplement: mjad006_Supplemental_File [file mjad006_supplemental_file.pdf]

Supplementary material

CircRNP complexes: from nature to design

Stephen Sukumar Nuthalapati, Corinna Jessica Ulshöfer, and Albrecht Bindereif*

Institute of Biochemistry, Justus Liebig University of Giessen, 35392 Giessen, Germany

* Correspondence to: Albrecht Bindereif, E-mail: albrecht.bindereif@chemie.bio.uni-giessen.de

Supplementary Materials and methods

In vitro synthesis of circRNA-based IMP3 sponge

Previous work from our lab had revealed an array of IMP3 RNA-binding motifs, based on SELEX analysis (Schneider et al., 2019). A natural high-affinity target, exon 29 of human ANKRD17, which is processed to a circRNA (Schneider et al., 2016), contains this RNA-binding array within 121 nucleotides; this was taken as a comparison to a synthetic, SELEX-based 101-mer RNA (Schneider et al., 2019). In a control sequence, the five elements were mutated to UG-repeats (**Figure 1A**). Each of these templates was synthesized by T7 transcription, using synthetic double-stranded oligonucleotides (Sigma-Aldrich) and PCR-extension for adding the 20-nucleotide stem-loop sequence (**Figure 1A**) and the T7 promoter.

For ^{32}P-labeling during *in vitro* transcription, RNA was synthesized with equal amounts of ATP, GTP and CTP (0.5 mM each), 0.04 mM UTP and 10 µCi of [α-^{32}P]-UTP. In addition, 10 mM DTT and a 4-fold excess of GMP (2 mM) were used. Following RQ1 DNase treatment, RNA was purified with Mini Quick Spin RNA columns (Roche). Transcripts were ligated with T4 RNA ligase at 16°C overnight, followed by gel extraction (Costar Spin-X centrifuge tube filters; Corning) and ethanol precipitation.

Expression and purification of recombinant human IMP3 protein

Recombinant human GST-/His-tagged IMP3 protein was expressed in *E. coli* BL21 cells, using Ni-NTA agarose, elution by imidazole, and dialysis (20 mM Tris-HCl pH 7.8, 100 mM KCl, 0.2 mM EDTA, 20% (*v*/*v*) glycerol, 1 mM DTT) (Schneider et al., 2019). For quantification of purified IMP3, analysis by SDS-PAGE and a BSA standard was used (**Figure 1B**).

Analysis of RNA binding of IMP3 protein by electrophoretic mobility shift assays (EMSA)

EMSA assays were performed with purified, recombinant IMP3 and ^{32}P-labeled linear or circular RNA. Binding reactions (30 min at 25°C, followed by placing on ice for 5 min) were done in binding buffer (10 mM Tris-HCl, pH 7.5, 150 mM NaCl, 0.5 mM EDTA, 0.5 mM DTT, 0.1% NP-40, 5% glycerol, supplemented with RNaseOUT, as well as 1 µg tRNA and 1 µg BSA as nonspecific competitors), and contained 5 nM of labelled RNA and IMP3 at concentrations ranging from 0–40 nM (ANKRD17, 101-mer) or 0–160 nM (all-UG mutant). Binding reactions were analyzed on native 5% TBE gels (containing 5% glycerol), pre-run for 20 min. Gel electrophoresis was performed for 45 min with 45 mA at 4°C. Radioactive signals were visualized by the Typhoon FLA 9500 Phosphorimager system and intensities quantified. Curve fitting of raw data used the quadratic binding equation (Altschuler et al., 2013), and for K_d calculations from experimental triplicates, OriginPro was employed (OriginLab) (**Figure 1C**).

RNA design based on SELEX-derived data

Highly specific, SELEX-derived RNAs were designed, based on motif enrichment and spacing analyses (for a detailed description of SELEX-seq analysis, see Schneider et al., 2019). The L_12/10/12 RNA (65 nt) contains four RNA-binding motifs (ACAC, ACAC, ACAU, and ACAU) for binding to the four RRM domains of hnRNP L, with a 10- or 12-nucleotide spacing in between (**Figure 2A**). As a negative control (mut L_12/10/12 RNA), ACAC was mutated to UGUG, ACAU to UGUU. RNAs were transcribed by T7 RNA polymerase and used in linear form, or circularized by T4 RNA ligase, followed by gel purification.

RNA transfection in HeLa cells and splicing analysis.

HeLa cells were cultured in DMEM medium supplemented with 10% FBS at 37°C in an incubator with 5% CO_2. For RNA transfection, 5×10^4 cells per well were seeded in a 24-well plate 24 h before transfection. 1 µg of gel-purified circRNA (or corresponding linear RNA) was transfected with Lipofectamine 2000 Invitrogen) in OptiMEM medium (Gibco, 3 µl/µg of RNA), according to the manufacturer’s instructions. Cells were harvested 24 h after transfection, and total RNA was extracted.

Cells were lysed (TRIzol, Ambion), and contaminating DNA was removed by RNase-free RQ1 DNase. Total RNA was extracted, and its concentration was measured at 260 nm (Nanodrop).

Reverse transcription (RT) was carried out by qScript™ Flex cDNA synthesis kit [Quanta Biosciences; random hexamers and oligo(dT) primers]. The reverse-transcription reaction was followed by PCR (with gene-specific primers; see Schreiner et al., 2020) and analysis on a 2% agarose gel by ethidium bromide staining (**Figure 2B**). Band intensities were quantified using ImageJ software.

Supplementary References

Altschuler, S.E., Lewis, K.A., and Wuttke, D.S. (2013). Practical strategies for the evaluation of high-affinity protein/nucleic acid interactions. J. Nucleic Acids Investig. *4*, 19-28.

Schneider, T., Hung, L.H., Schreiner, S., et al. (2016). CircRNA-protein complexes: IMP3 protein component defines subfamily of circRNPs. Sci. Rep. *6*, 31313.

Schneider, T., Hung, L.H., Aziz, M., et al. (2019). Combinatorial recognition of clustered RNA elements by the multidomain RNA-binding protein IMP3. Nat. Commun. *10*, 2266.
